# Throughput Measurement of a Dual-Band MIMO Rectangular Dielectric Resonator Antenna for LTE Applications

**DOI:** 10.3390/s17010148

**Published:** 2017-01-13

**Authors:** Jamal Nasir, Mohd. Haizal Jamaluddin, Aftab Ahmad Khan, Muhammad Ramlee Kamarudin, Chee Yen Leow, Owais Owais

**Affiliations:** 1 Wireless Communication Centre, Universiti Teknologi Malaysia, 81310 UTM Skudai, Johor, Malaysia; jamalnasir@ciit.net.pk (J.N.); ramlee@fke.utm.my (M.R.K.); bruceleow@fke.utm.my (B.L.C.Y.); 2 Department of Electrical Engineering, COMSATS Institute of Information Technology, 22060 Abbottabad, Pakistan; aftabjadoon@ciit.net.pk (A.A.K.); mkowais@ciit.net.pk (O.O.)

**Keywords:** dielectric resonator antenna, MIMO, LTE, mutual coupling

## Abstract

An L-shaped dual-band multiple-input multiple-output (MIMO) rectangular dielectric resonator antenna (RDRA) for long term evolution (LTE) applications is proposed. The presented antenna can transmit and receive information independently using fundamental TE_111_ and higher order TE_121_ modes of the DRA. TE_111_ degenerate mode covers LTE band 2 (1.85–1.99 GHz), 3 (1.71–1.88 GHz), and 9 (1.7499–1.7849 GHz) at *f_r_ = 1.8* GHz whereas TE_121_ covers LTE band 7 (2.5–2.69 GHz) at *f_r_ = 2.6* GHz, respectively. An efficient design method has been used to reduce mutual coupling between ports by changing the effective permittivity values of DRA by introducing a cylindrical air-gap at an optimal position in the dielectric resonator. This air-gap along with matching strips at the corners of the dielectric resonator keeps the isolation at a value more than 17 dB at both the bands. The diversity performance has also been evaluated by calculating the envelope correlation coefficient, diversity gain, and mean effective gain of the proposed design. MIMO performance has been evaluated by measuring the throughput of the proposed MIMO antenna. Experimental results successfully validate the presented design methodology in this work.

## 1. Introduction

LTE developed by third generation partnership project (3GPP) is a fourth generation (4G) wireless standard that provides 10 times the speed of 3G network [1]. It is designed to provide IP-based data, voice, and multimedia streaming at a speed ranging from 100 Mbps to 1 Gbps. Because of this dramatic increase in capacity and speed of data transmission, multiple antenna techniques have gained overwhelming effects in the area of wireless communication throughout the last decade. However, designing of antennas for such a high-speed data transmission poses challenges in terms of isolation between antenna elements and the size of the antenna which may ultimately affect system performance. A considerable amount of work has already been done in the area of microstrip patch antennas (MPAs) but they suffer from low bandwidth [2] and metallic losses at high frequencies.

Dielectric resonators when used as a radiator have low losses, high radiation efficiency, and high bandwidth as compared to the conventional MPAs [3]. Importantly, various modes can be excited with diverse radiation characteristics using a single dielectric resonator which makes it an appropriate alternative for multifunction applications [4].

Ishimiya et al. for the first time gave the concept of a MIMO DRA and achieved 10 dB diversity gain [5] which is comparable with MIMO dipole array used in IEEE 802.11n, but no explicit design method is given in this paper. A dual-port MIMO design at 700 MHz for LTE Femtocell base station applications is proposed in [6] that achieved channel capacity up to 11.1 bps/Hz with excellent port isolation. In [7], the authors proposed a single element dual-port MIMO rectangular DRA at 2.6 GHz excited by two symmetric microstrip feed lines. Acceptable results have been obtained in terms of S-parameters, radiation pattern plots, correlation coefficient, mean effective gain, and diversity gain. In [8], the authors proposed rectangular DRA at 2.6 GHz, excited by coplanar waveguide and a coaxial probe and obtained reasonable results in terms of correlation coefficient and diversity gain.

Antennas proposed in [5,6,7,8] are designed for single-band MIMO operation. A lot of research studies have been presented on dual-polarized microstrip patch antennas but, their metallic losses at high frequencies become substantial. A compact and multiband DRA for mobile handheld devices for DVB-H, Wi-Fi, and WiMAX is presented in [9] using two dielectric resonators. However, there is large ground size and high mutual coupling values, especially at the higher band. Another dual-band DRA with MIMO characteristics reported in literature is in cylindrical form covering DCS and WLAN bands [10]. This design has good isolation but with large values of ground plane and DRA height.

In this paper, a MIMO rectangular DRA with dual-band characteristics is reported using a single radiator to operate at 1.8 and 2.6 GHz bands for LTE applications. A rectangular shape is chosen because it has one more degree of freedom than cylindrical and two degrees of freedom than hemispherical DRAs. Lower frequency band of the proposed design may be termed as wideband as it covers three LTE bands 2, 3, and 9 simultaneously. The simulation of the presented MIMO antenna is performed with the help of Ansoft HFSS v 16.0 and a good agreement is found between the simulated and measured results.

The remainder of this paper covers antenna geometry description in Section 2. Antenna design analysis including effect of electric field on mutual coupling will be presented in Section 3. Section 4 is dedicated to discuss results in detail and Section 5 concludes the paper.

## 2. Geometry of the Presented Antenna

**Figure 1:**
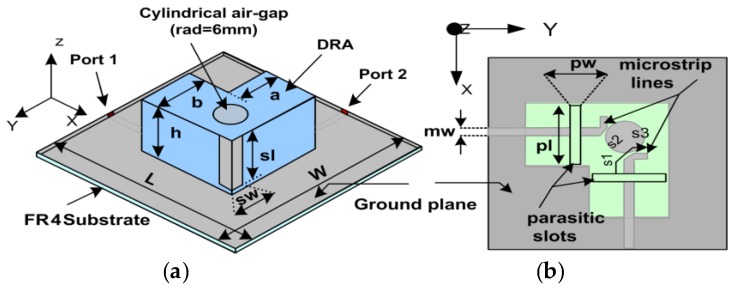
Presented antenna Geometry (**a**) 3-D view; (**b**) Top view.

The geometry of the proposed design is shown in Figure 1. It consists of an L-shaped rectangular dielectric resonator (DR) excited by two symmetrical slots of equal dimensions coupled through microstrip feed lines. DR is made up of ceramic material (ε_r_ = 10) and has a loss tanδ = 0.002, and is placed on a ground plane of size 100 × 100 mm^2^. FR4 substrate (ε_r_ = 4.6) of the size of the ground plane with height 1.6 mm is the base layer of the design. Figure 1a shows a cylindrical air-gap of 22 mm in height with a hole of 6 mm radius drilled in the DR along with two metallic strips (6 × 20 mm^2^) at the optimized position to improve isolation. A top view of the design is shown in Figure 1b, in which *pl* and *pw* is the length and width of both the coupling slots, whereas *s1 + s2 + s3* is the stub length to improve impedance matching and m_w is the width of the microstrip feed lines (placed at the bottom surface of the substrate). Both the coupling slots have the dimensions *pw* × *pl* etched on the ground plane and are fed by two 50 Ω microstrip feed lines. Table 1 lists the optimized dimensions of the proposed design after a thorough parametric analysis.

## 3. Antenna Design and Analysis

This section presents the details about the dimension calculation of the DR and the parametric study of the design along with the effects of the cylindrical air-gap and metal strips on the mutual coupling between the ports.

### 3.1. Initial DR Dimensions and Parametric Study

**Figure 2:**
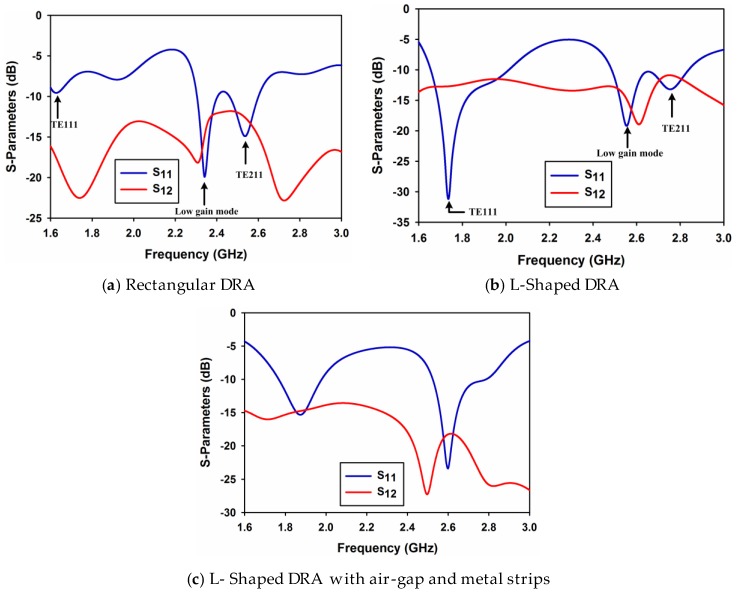
S-Parameters Plots.

Initial values of the DRA dimensions are calculated using dual-band formulas given in [10]. While calculating the dimensions of the proposed dual-band DRA, all the equations given in [10] for dual band DRAs were used. Only the equations for kx1 and kx2 for TE^y^_111_ and TE^y^_211_ and ky1 and ky2 for TE^x^_111_ and TE^x^_121_ modes were modified because the excited higher order modes in the presented DRA are TE^y^_111_ and TE^y^_211_, TE^x^_111_ and TE^x^_121_ as compared to the higher order mode excited in [10] which is TE^y^_113_. The modified values are given in Equations (1) and (2).

(1) $$Kx1=\frac{\pi }{a},\;Kx2=\frac{2\pi }{a}\;and\;Kz1=\frac{\pi }{2h},\;Kz2=\frac{\pi }{2h}$$

(2) $$Ky1=\frac{\pi }{b},\;Ky2=\frac{2\pi }{b}\;and\;Kz1=\frac{\pi }{2h},\;Kz2=\frac{\pi }{2h}$$

where kx, ky, and kz are the wave numbers in the x, y, and z direction, respectively. The design formulas given in [10] are applicable to a rectangular shaped DRA only. Therefore, based on these formulas, the dimensions of the dual-band rectangular DRA were obtained. The DRA was simulated and corresponding S-parameters are shown in Figure 2a. S-parameters from port 2 are not shown as they are similar to port 1. It is clear from this figure that there are three resonances at 1.68 GHz, 2.33 GHz, and 2.54 GHz. The first resonance relates to TE111 mode with poor matching while the second resonance is due to some unknown higher order mode at 2.33 GHz with low simulated gain (3.8 dBi). The third resonance is because of the TE121 mode. In order to improve matching and shift the resonance frequencies to the required bands of interest, the size of the DRA needs to be changed. As the modes excited are TE^x^ and TE^y^, the resonance frequency of the excited modes is sensitive to the dimension of the DRA along x and y-axis. Also, to maintain symmetry between the ports along with change in size in the x and y-axis, the rectangular shape was modified to an L-shaped DRA. Doing so, the size of the DRA has been modified in the x and y-axis (dimension a and b) which in turn changes the wave numbers of kx and ky, thus shifting the lower resonance frequency towards the higher band while merging the two resonances at the upper band to give a wide band. The S-parameters of the L-shaped DRA are shown in Figure 2b. As evident from the figure, the resonance at the lowest frequency has shifted to 1.8 GHz while at the upper frequency band, the two resonances have merged to obtain a wideband and matching has been improved. However, isolation between the ports at the bands of interest is poor. To improve port isolation, cylindrical air-gap and metal strips have been incorporated in the L-shaped DRA and the results are shown in Figure 2c.

**Figure 3:**
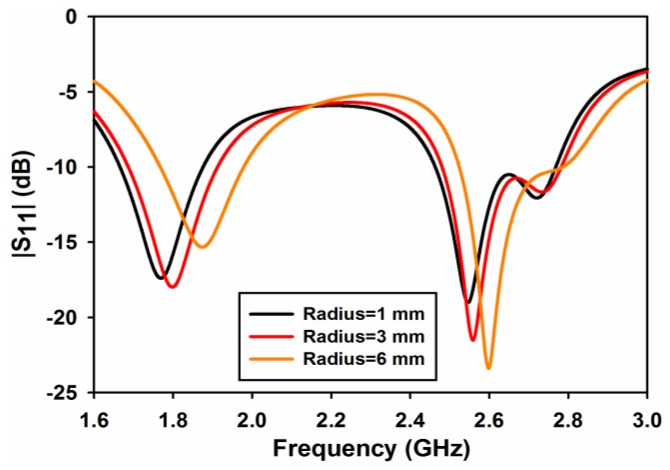
|S_11_| for different values of cylinder radius.

**Figure 4:**
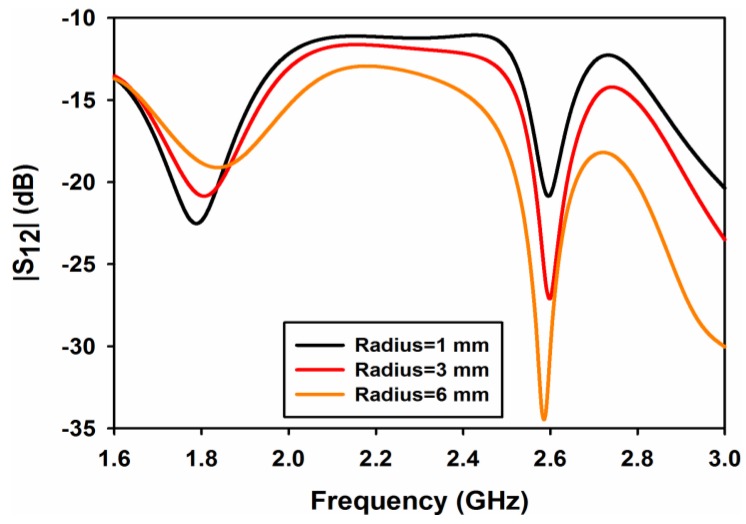
|S_12_| for different values of cylinder radius.

A parametric study on cylindrical air-gap radius *rad* and length of metallic strips *sl* is performed to evaluate the performance of the design. Figure 3 shows the effect of the cylindrical air-gap radius *rad* on reflection coefficient of port 1 (S_11_). As both ports 1 and 2 are symmetrical, only the results of port 1 are shown in the parametric study. As can be observed from the figure that the radius of the cylindrical air-gap has a little effect on reflection coefficient. A minor shift in frequency can be observed in Figure 3 at both frequency bands. Similarly, the effect of the cylindrical air-gap radius on transmission coefficient (S_12_ or S_21_) is shown in Figure 4. It is clearly observable from this figure that, by increasing the radius of the air-gap, the isolation at 2.6 GHz improves considerably and its value reaches almost to 35 dB for rad = 6 mm. At 1.8 GHz, the isolation decreases with the increase in *rad* but at a very low rate and has a value of 13 dB at *rad* = 6 mm.

**Figure 5:**
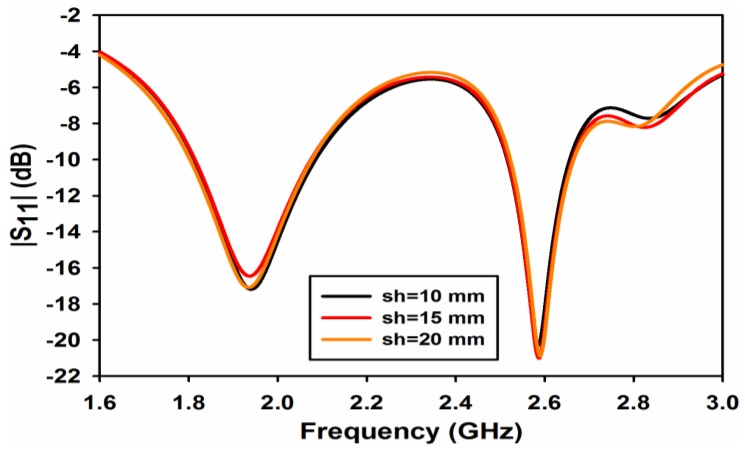
|S_11_| for different value of sheet height.

**Figure 6:**
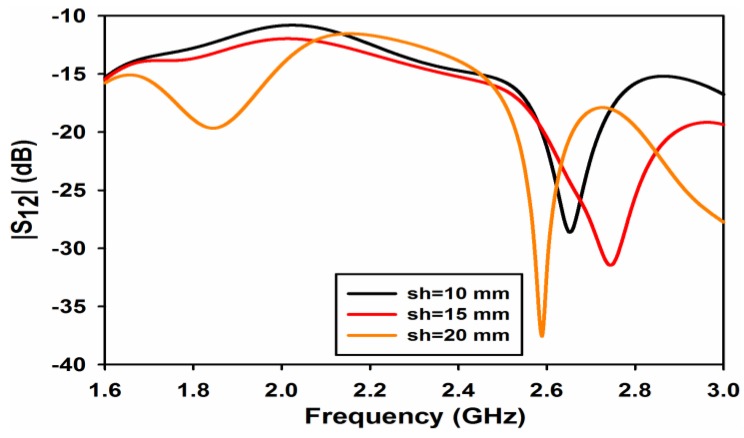
|S_12_| for different value of sheet height.

The effect of the metallic sheet strip length *sl* on the reflection and transmission coefficients is shown in Figure 5 and Figure 6, respectively. From Figure 5, it is quite apparent that the length of the strip has negligible effect on the reflection coefficient at both the frequency bands. A considerable effect of *sl* can be observed on the isolation of the ports as shown in Figure 6. The isolation at 1.8 GHz for *sl* = 10 and 15 mm is almost 12 and 13 dB while the isolation improves to 19 dB when the strip length increases to 20 mm. At the 2.6 GHz band, the length of the strip shifts isolation depth towards the lower frequency giving a value of isolation of 38 dB at 2.6 GHz for a value of *sl* = 20 mm.

### 3.2. Electric Field Effects in DR

**Figure 7:**
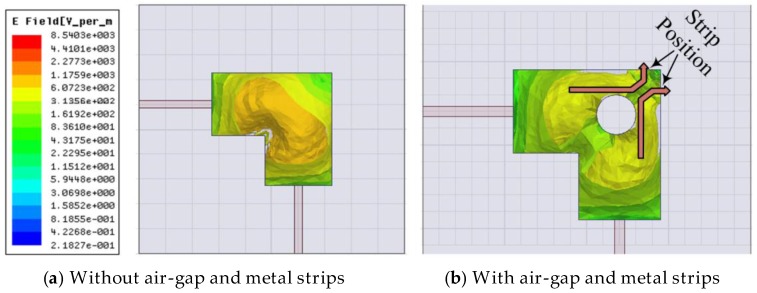
E-field Magnitude plot.

If we analyze the effects of electric field in the DR, it clearly demonstrates the reason of mutual coupling reduction by using the cylindrical air-gap and the metal strips. The electric fields originating from both the ports interact with each other and are a cause of mutual coupling in the DR. This interaction between the E-fields is reduced by using a cylindrical air-gap and a pair of metal strips. At the field intersection point, a cylindrical air-gap is introduced which changes the corresponding phases of the E-field at the boundaries of the air-gap. When the field enters from a higher to lower primitively material cylindrical air-gap, it deviates away from the normal direction [11], thus reducing the interaction between the E-field from the two ports. This effect can be seen at the boundaries where field through each port is tangentially oriented resulting in a considerable decrease in mutual coupling. In order to further reduce the coupling effects, two metallic strips are introduced at the corner of the DR shown in Figure 1a. It is well known that the tangential components of electric field vanish when it strikes on a metallic surface, only the transverse component can exist. This results in further deviation of the E-filed originating from each port from each other resulting in a further reduction in mutual coupling. Air-gaps [12] and metallic strips are being used to increase bandwidth and impedance matching, respectively. However, in this design this combination has been used as a coupling reduction technique. The magnitude E-field plot is shown in Figure 7.

## 4. Antenna Performance Measurements

This section includes the antenna and MIMO performance parameters. First, the antenna performance parameters such as S-parameters, total efficiency, and radiation patterns are described; followed by diversity and MIMO performance matrices like ECC, MEG, DG, and channel capacity (throughput) calculation.

### 4.1. Antenna Parameters Analysis

**Figure 8:**
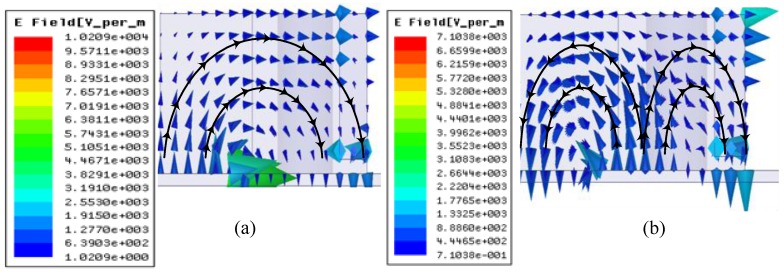
E-field Distribution for (**a**) TE^x^_δ1_ (**b**) TE^x^_δ21_.

**Figure 9:**
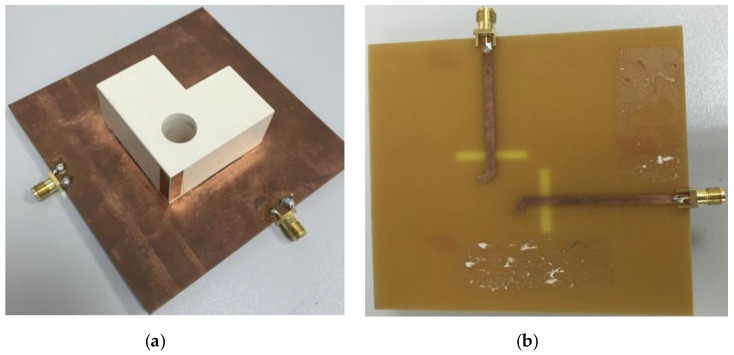
Prototype of the proposed MIMO antenna (**a**) 3-D view (**b**) Bottom view.

**Figure 10:**
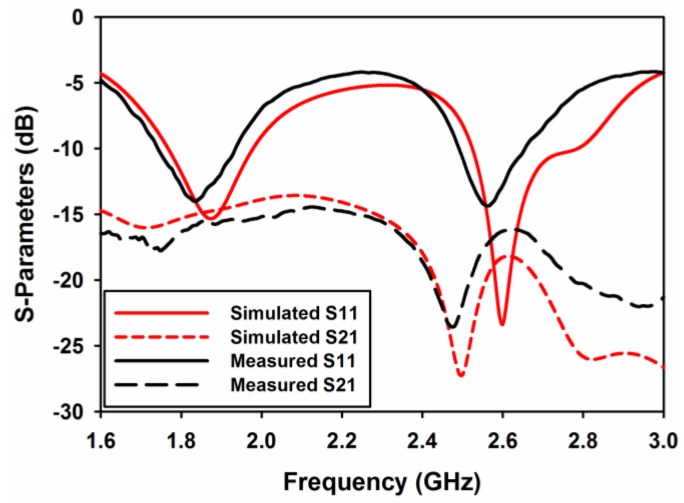
Measured and simulated S-parameters.

For the antenna to be resonating at 1.8 and 2.6 GHz, two orthogonal modes have to be excited simultaneously. Port 1 excites dominant TE^x^_δ11_ and higher order TE^x^_δ21_ modes whereas port 2 excites TE^y^_1δ1_ and TE^y^_2δ1_ modes, respectively. Because of the symmetry of both ports, the E-field distribution for both modes is shown in Figure 8 for port 1 only. The prototype of the proposed antenna is shown in Figure 9. The ground plane is etched on FR4 substrate and the DR is made up of Eccostock HK-10 material with tanδ = 0.002. Microstrip feed lines are placed at the opposite side of the substrate to feed DR through two slots of equal dimensions. Figure 10 demonstrates the comparison of both the simulated and measured S-parameters. S-parameters for reference level −10 dB are measured.

Measured fractional bandwidth at 1.8 GHz is 18% (1.71–2.05 GHz) and at 2.6 GHz is 8% (2.5–2.7 GHz) through ports 1 and 2, respectively. Figure 10 indicates a good agreement between measured and simulated results. The antenna is well matched at both resonances, covering four LTE bands as mentioned earlier.

Simulated total radiation efficiencies through ports 1 and 2 are the same i.e., 94.42% at 1.8 GHz and 96% at 2.6 GHz, respectively. These values are calculated using Equations (3) and (4) [13].

(3) $$\mu_{1,tot}=\;\mu_{1,rad}\left(1-{\left|s_{11}\right|}^{2}-{\left|s_{21}\right|}^{2}\right)$$

(4) $$\mu_{2,tot}=\;\mu_{2,rad}\left(1-{\left|s_{22}\right|}^{2}-{\left|s_{12}\right|}^{2}\right)$$

**Figure 11:**
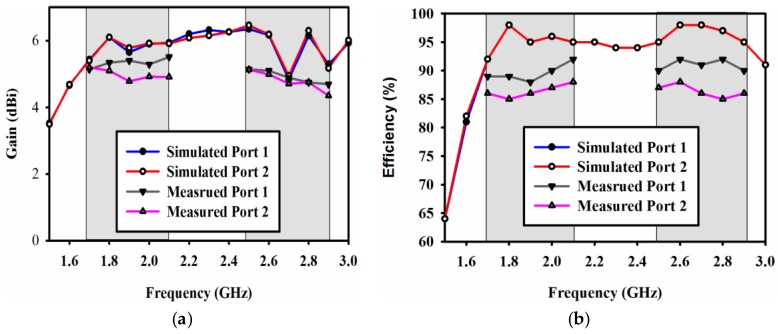
Simulated and measured (**a**) Gain; (**b**) Efficiency.

Figure 11 shows the simulated and measured gain and efficiency plots of the proposed MIMO DRA. The measured gain and efficiency is shown only at the bands of interest. It can be easily seen that the measured gain at 1.8 GHz for ports 1 and 2 is 5.5 dBi and 5.2 dBi, respectively. While at 2.6 GHz, the measured gain for ports 1 and 2 is 5.4 dBi and 5.3 dBi, respectively. Similarly, the measured efficiency at 1.8 GHz for ports 1 and 2 is 90% and 89% while at 2.6 GHz for ports 1 and 2 is 93% and 91%, respectively.

**Figure 12:**
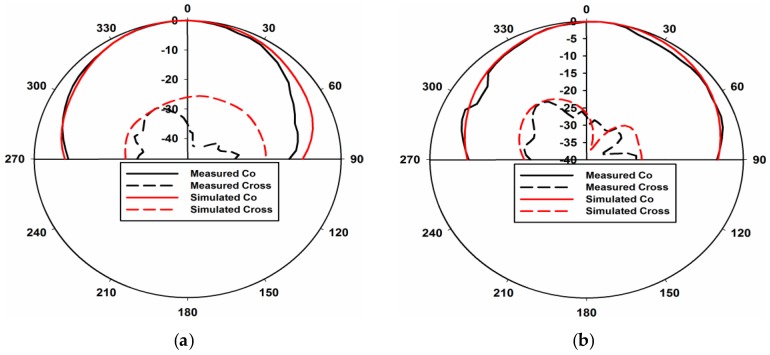

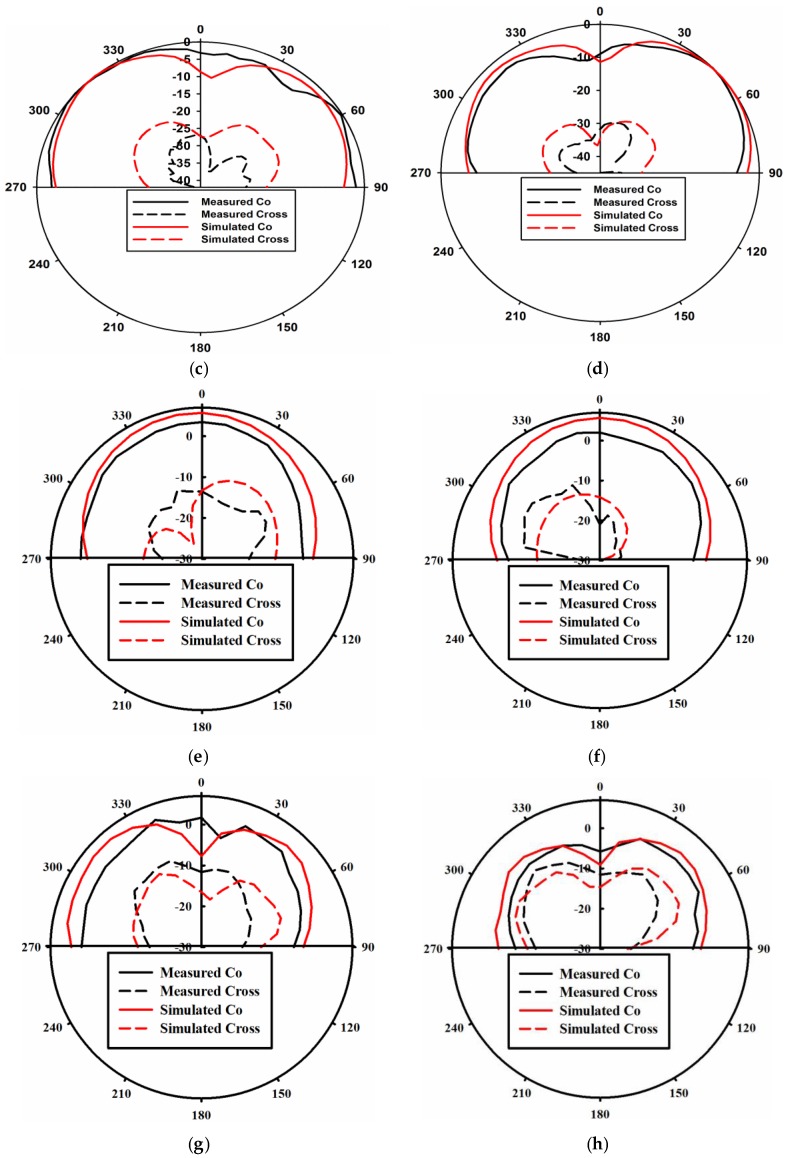
Measured and simulated radiation patterns. (**a**) E-Plane Port 1 at 1.8 GHz; (**b**) E-Plane Port 2 at 1.8 GHz; (**c**) E-Plane Port 1 at 2.6 GHz; (**d**) E-Plane Port 2 at 2.6 GHz; (**e**) H-Plane Port 1 at 1.8 GHz; (**f**) H-Plane Port 2 at 1.8 GHz; (**g**) H-Plane Port 1 at 2.6 GHz; (**h**) H-Plane Port 2 at 2.6 GHz.

**Figure 13:**
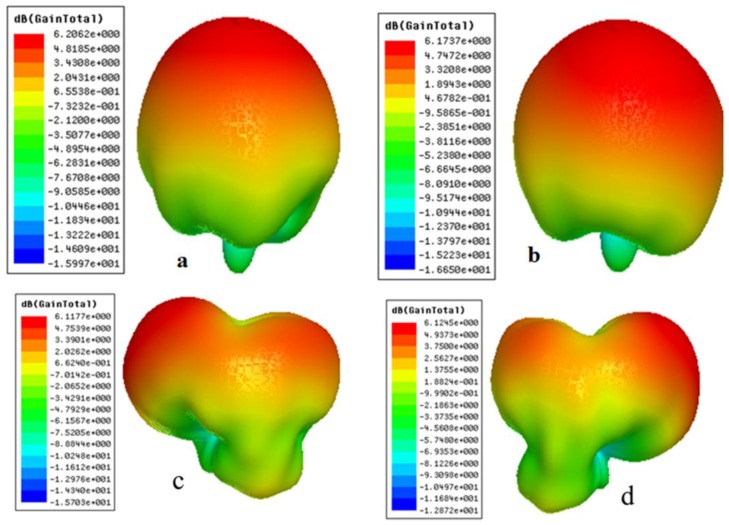
3-D Simulated Radiation Patterns. (**a**) Port 1 at 1.8 GHz; (**b**) Port 2 at 1.8 GHz; (**c**) Port 1 at 2.6 GHz; (**d**) Port 2 at 2.6 GHz.

Figure 12a–d shows E-plane views and Figure 12e–h shows the H-plane views of the measured and simulated radiation patterns at 1.8 and 2.6 GHz through ports 1 and 2, respectively. Radiation patterns shown in Figure 12a,b point in broadside directions for both ports 1 and 2 at 1.8 GHz. Similarly, radiation patterns shown in Figure 12 c,d point in +45° and 315° for ports 1 and 2 at 2.6 GHz, respectively. The cross polarization values in all the plots are well below the co polarization values. The same is true for the H-plane patterns. These results clearly indicate pattern diversity operation of the presented MIMO antenna. The 3-D radiation pattern plots of the proposed dual-band MIMO antenna are presented in Figure 13. The 3-D patterns are shown for both the ports at 1.8 GHz and 2.6 GHz, respectively.

### 4.2. Diversity Performance Analysis

**Figure 14:**
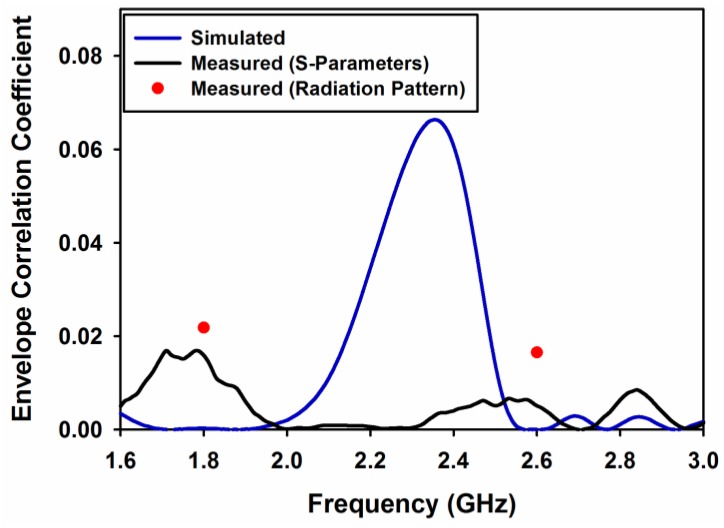
Measured and simulated ECC.

Envelope correlation coefficient (ECC) is the first diversity and MIMO parameter to be analyzed. It gives a correlation between signals at the receiving end. In this work, ECC is calculated by using both S-parameter using Equation (5) and 3D radiation pattern using Equation (6) [14]. Simulated and measured ECC using S-parameters Equation (5) are shown in Figure 14.

(5) $$\rho_{e}=\frac{{\left|S_{11}^{*}S_{12}+S_{21}^{*}S_{22}\right|}^{2}}{\left(1-\left({\left|S_{11}\right|}^{2}+{\left|S_{21}\right|}^{2}\right)\right)\left(1-\left({\left|S_{22}\right|}^{2}+{\left|S_{12}\right|}^{2}\right)\right)}\;$$

(6) $$\rho_{e}=\;\frac{{\left|ʃ{ʃ}_{4\pi }F_{1}^{\to^{*}}\left(\theta ,\varphi \right)\;.\;F_{2}^{\to^{*}}\left(\theta ,\varphi \right)dΩ\right|}^{2}}{ʃ{ʃ}_{4\pi }{\left|F_{1}^{\to^{*}}\left(\theta ,\varphi \right)\right|}^{2}dΩʃ{ʃ}_{4\pi }{\left|F_{2}^{\to^{*}}\left(\theta ,\varphi \right)\right|}^{2}dΩ}\;$$

ECC using far field pattern Equation (6) is found to be 0.0218 and 0.0165 at 1.8 and 2.6 GHz, respectively and is shown as red dots in Figure 14. This figure also shows that ECC values are well below 0.5 in the desired frequency bands, which ensures good diversity performance [15].

Diversity gain (DG) is another import parameter which assures good diversity and MIMO performance. In this work, DG is calculated using Equation (7) [8].

(7) $$DG=10e_{\rho }$$

where

(8) $$e_{\rho }=\sqrt{\left(1-{\left|0.99\rho_{e}\right|}^{2}\right)}$$

**Figure 15:**
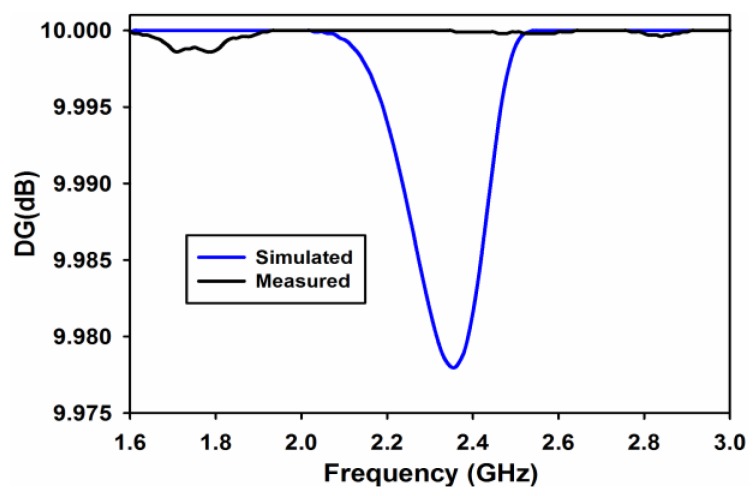
Measured and simulated diversity gain.

Figure 15 shows the measured and simulated diversity gain of the proposed dual-band MIMO antenna. The diversity gain in the both the frequency bands is almost 10 dB.

Mean effective gain (MEG) is the last and very important parameter to be analyzed for diversity performance. It is defined as the ratio of the average power received at the antenna to the sum of the average power of the vertically and horizontally polarized waves received by isotropic antenna [16]. Using S-parameters MEG for each port, it can be measured using Equation (9) [17]. For similar power levels of each branch, power ratio k, which is equal to the difference in the magnitude of MEGs, is calculated using Equation (10) [18].

(9) $$MEG_{i}=0.5\eta_{i,rad}=0.5\left[1-\overset{M}{\underset{j=1}{\sum }}|S_{ij}{|}^{2}\right]\;$$

(10) $$k=\left|MEG_{1}-MEG_{2}\right|<3dB$$

**Figure 16:**
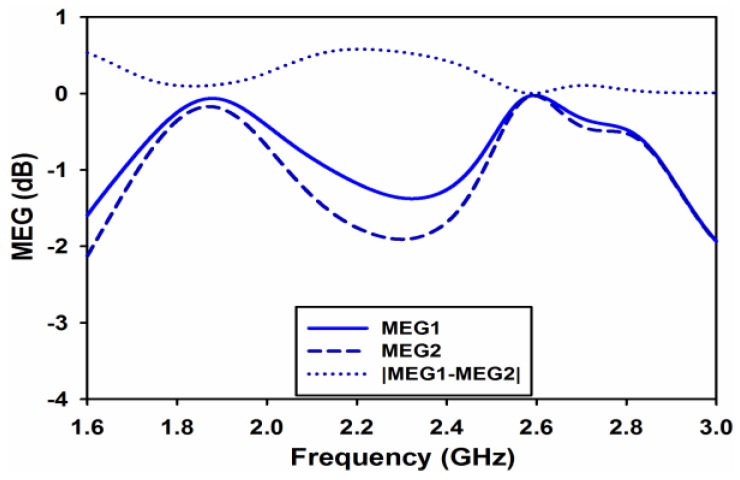
Simulated Mean Effective Gain (MEG).

**Figure 17:**
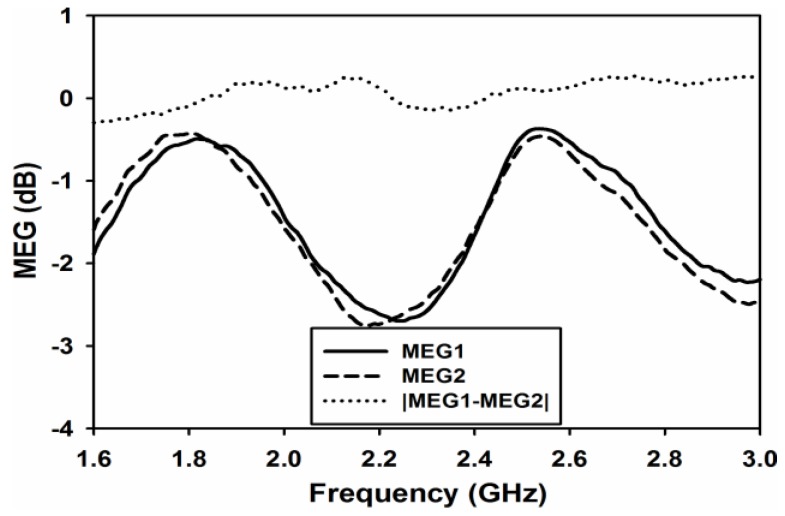
Measured Mean Effective Gain (MEG).

Figure 16 and Figure 17 shows the simulated and measured MEGs and both the figures clearly show that k is almost equal to 0 at both the desired frequency bands.

### 4.3. MIMO Performance Analysis

**Figure 18:**
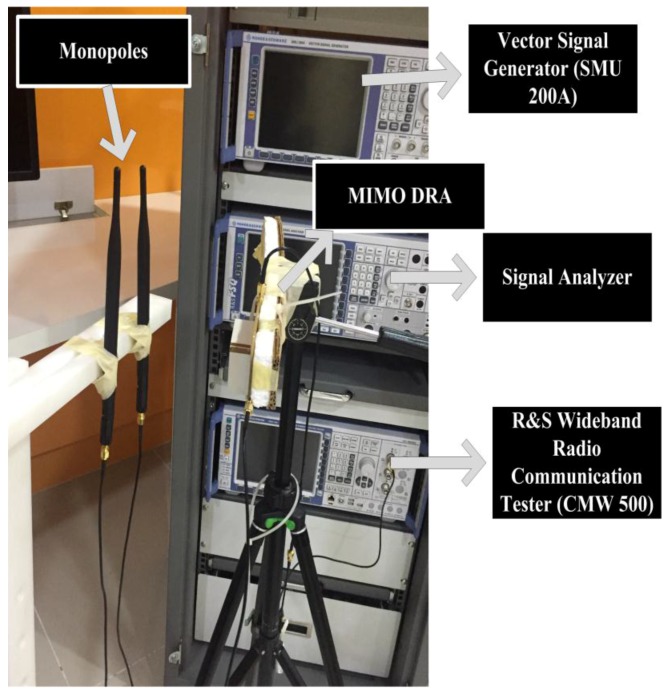
Channel capacity measurement setup.

This section presents the MIMO performance analysis of the proposed dual-band MIMO DRA. MIMO performance in terms of data throughput has been measured. For the throughput measurements of the proposed design, the setup shown in Figure 18 has been used. The procedure presented in [19] has been used in this paper for the throughput measurements. Throughput, which is measured in bits per second (bps) in a communication network, is the amount of data transferred per unit time through a communication link from source to destination. The aggregate or system throughput is the overall data rates transferred to all the destinations in a communication network. The term throughput is often used for maximum throughput. The maximum throughput (bits/sec or bps) of a communication link or a node is also termed as its capacity. Figure 18 shows the MIMO throughput measurement setup. The figure shows the proposed dual-band MIMO antenna along with two monopoles (separated by 0.5λ spacing). The separation between the proposed DRA and monopole is 30 cm. Both the antennas are connected to Wideband Radio Communication Tester R&S^®^CMW500 (LTE node B (eNodeB)) along with a Huawai E398 USB modem dongle (which acts as the LTE user equipment (UE)). By using dedicated Physical Downlink Shared Channel sub frames the eNodeB (CMW500), the emulator transmits downlink data to the UE. The throughput is calculated using the Block Error Rate (BLER). The positive and negative acknowledgments returned by the UE are used to determine BLER. The throughput measurements of the given MIMO antenna were performed for a SISO and 2 × 2 MIMO systems. Three types of modulations including QPSK, 16 QAM (quadrature amplitude modulation), and 64 QAM have been used to measure the throughput for the SISO and MIMO system of the presented antenna. Table 2 and Table 3 depict the measured throughput for the SISO and MIMO antenna systems for the three modulation schemes along with the specified channel quality indicator (CQI) at 1.8 GHz and 2.6 GHz, respectively. The parameters used in the measurement setup include: bandwidth of 20 MHz, 100 number of resource blocks, −20 dBm of transmitted power, frequency bands of 1.8 GHz (band 3), and 2.6 GHz (band 7).

From Table 2 and Table 3, it can be clearly seen that the measured throughputs at both the frequency bands are close to the maximum throughput. At 1.8 GHz for the SISO case, the proposed MIMO DRA achieves 99.9% of the theoretical maximum throughput for all modulation schemes. For the case of MIMO at the same frequency, the presented antenna achieves 99.9% of the theoretical maximum throughput for all modulation schemes. Similarly, at 2.6 GHz for all the modulation schemes, the presented MIMO DRA achieves 99.9% of the maximum theoretical throughput for both SISO and MIMO scenario.

All the results analyzed in this work clearly indicate that the proposed antenna is suitable for LTE applications and it is able to deliver an excellent throughput performance. The 2 × 2 MIMO performance also justifies that the proposed antenna has a good level of isolation between ports to support the MIMO spatial multiplexing operation.

## 5. Conclusions

A dual-band dual-port MIMO RDRA fed by two symmetric coupling apertures is proposed and investigated. Two orthogonal radiating modes are excited in the DRA simultaneously at two overlapping frequency ranges around 1.8 and 2.6 GHz. The measured isolation is better than 17 dB over the operating frequency ranges. Given the same frequencies and same dielectric material, the proposed antenna is the first with a rectangular shape to be operated at given frequencies with such a high isolation values at both the bands for MIMO applications. ECC calculated from S-parameters and 3-D radiation pattern is well below 0.5 which is the required acceptable level. Measured DG is almost 10 dB and MEG ratios are maintained close to zero at both bands. The throughput measurement at both the frequency bands indicates superior performance to an SISO system in terms of more bits transferred per second. The presented results prove that the proposed antenna can provide considerable performance for LTE MIMO applications.

## Figures and Tables

**Table 1:** Presented design optimized dimensions.

Parameter	Value (mm)	Parameter	Value (mm)	Parameter	Value (mm)
*a*	20	b	25	*h*	22
*sl*	20	sw	6	*pl*	23
*pw*	3	s1	5.25	*s2*	3
*s3*	4.5	mw	3	*rad*	6

**Table 2:** Measured throughput with three modulation schemes at 1.8 GHz.

Modulation Scheme (CQI)	Measured Throughput SISO	Measured Throughput MIMO	Maximum Throughput SISO	Maximum Throughput MIMO
**QPSK(Mbps) (6)**	14.10	28.21	14.112	28.23
**16QAM (Mbps) (9)**	30.33	56.65	30.352	56.68
**64QAM (Mbps) (12)**	50.190	93.152	50.197	93.161

**Table 3:** Measured throughput with three modulation schemes at 2.6 GHz.

Modulation Scheme (CQI)	Measured Throughput SISO	Measured Throughput MIMO	Maximum Throughput SISO	Maximum Throughput MIMO
**QPSK(Mbps) (6)**	14.11	28.22	14.112	28.23
**16QAM (Mbps) (9)**	30.350	56.66	30.352	56.68
**64QAM (Mbps) (12)**	50.191	93.158	50.197	93.161
